# Therapeutic Potential of Diosgenin and Its Major Derivatives against Neurological Diseases: Recent Advances

**DOI:** 10.1155/2020/3153082

**Published:** 2020-03-06

**Authors:** Bangrong Cai, Ying Zhang, Zengtao Wang, Dujuan Xu, Yongyan Jia, Yanbin Guan, Aimei Liao, Gaizhi Liu, ChangJu Chun, Jiansheng Li

**Affiliations:** ^1^Henan Research Center for Special Processing Technology of Chinese Medicine, School of Pharmacy, Henan University of Chinese Medicine, Zhengzhou 450046, China; ^2^Department of Biochemistry, Department of Biomedical Sciences, Research Center for Aging and Geriatrics, Research Institute of Medical Sciences, Chonnam National University Medical School, Gwangju 501-190, Republic of Korea; ^3^Department of Medicinal Chemistry, College of Pharmacy JiangXi University of Traditional Chinese Medicine, Nanchang 330004, China; ^4^College of Biological Engineering, Henan University of Technology, Zhengzhou 450001, China; ^5^Research Institute of Drug Development, College of Pharmacy, Chonnam National University, Gwangju, Republic of Korea; ^6^Collaborative Innovation Center for Respiratory Disease Diagnosis and Treatment and Chinese Medicine Development of Henan Province, Henan Key Laboratory of Chinese Medicine for Respiratory Disease, Henan University of Chinese Medicine, China

## Abstract

Diosgenin (DG), a well-known steroidal sapogenin, is present abundantly in medicinal herbs such as *Dioscorea rhizome*, *Dioscorea villosa*, *Trigonella foenum-graecum*, *Smilax China*, and *Rhizoma polgonati*. DG is utilized as a major starting material for the production of steroidal drugs in the pharmaceutical industry. Due to its wide range of pharmacological activities and medicinal properties, it has been used in the treatment of cancers, hyperlipidemia, inflammation, and infections. Numerous studies have reported that DG is useful in the prevention and treatment of neurological diseases. Its therapeutic mechanisms are based on the mediation of different signaling pathways, and targeting these pathways might lead to the development of effective therapeutic agents for neurological diseases. The present review mainly summarizes recent progress using DG and its derivatives as therapeutic agents for multiple neurological disorders along with their various mechanisms in the central nervous system. In particular, those related to therapeutic efficacy for Parkinson's disease, Alzheimer's disease, brain injury, neuroinflammation, and ischemia are discussed. This review article also critically evaluates existing limitations associated with the solubility and bioavailability of DG and discusses imperatives for translational clinical research. It briefly recapitulates recent advances in structural modification and novel formulations to increase the therapeutic efficacy and brain levels of DG. In the present review, databases of PubMed, Web of Science, and Scopus were used for studies of DG and its derivatives in the treatment of central nervous system diseases published in English until December 10, 2019. Three independent researchers examined articles for eligibility. A total of 150 articles were screened from the above scientific literature databases. Finally, a total of 46 articles were extracted and included in this review. Keywords related to glioma, ischemia, memory, aging, cognitive impairment, Alzheimer, Parkinson, and neurodegenerative disorders were searched in the databases based on DG and its derivatives.

## 1. Introduction

With an increasingly aging population, human neurological disorders have become a great burden in terms of impact on quality of life and living costs [1]. In developed countries, a dramatic improvement in average life expectancy has led to substantial increases in the prevalence of diseases that mainly afflict the elderly [2, 3]. Human neurological disorders, including stroke, Alzheimer's disease, Parkinson's disease, depression, ischemic brain injury, and spinal cord injury, resulting from gradual and progressive loss of neural cells in CNS can lead to nervous system dysfunction [4]. Currently, no clinically effective treatments are available for these diseases. The only options to treat CNS disorders are by using drugs and performing surgery [5, 6]. However, most patients with neurological diseases need lifelong medication and long-term use of drugs is associated with serious side effects. Surgical treatment often increases the chance of infection and leads to other dysfunctions. In recent years, natural medicine has shown a great potential to treat nervous system disorders in many western countries [7, 8].

Natural products (NPs) derived from medicinal herbs, plants, vegetables, and fruits play an important role in the prevention and treatment of various human diseases, including cancer, cardiovascular disorders, diabetes, obesity, metabolic syndromes, and neurological disorders. NPs isolated from Chinese herbs have been widely used in traditional medicine over centuries. Many natural products derived from herbs exhibit a wide range of pharmacological properties, including the antimalarial drug artemisinin from *Artemisia apiacea* [9], the anticancer drug paclitaxel from *Taxus brevifolia* [10], and quercetin found in various vegetables and fruits [11]. Diosgenin (DG) is a naturally occurring steroidal sapogenin isolated from *Agavaceae*, *Dioscoreaceae*, *Liliaceae*, *Solanaceae*, *Scrophulariaceae*, *Amaryllidaceae*, *Leguminosae*, and *Rhamnaceae* [12–17]. It has been extensively studied for the management and treatment of different types of cancer [18], osteoporosis [19], cardiovascular diseases [20], atherosclerosis [21], diabetes mellitus [22], and skin diseases [23]. DG is being increasingly investigated in the treatment of neurological diseases [24]. Numerous studies have demonstrated that DG and its derivatives have preventive and therapeutic effects against various neurological disorders. Animal experiments have shown that DG is active in the treatment of nervous system diseases such as Parkinson's disease and Alzheimer's disease [25–27].

Despite its pharmacological activities in the treatment of various diseases, the clinical application of DG is severely hindered by its low aqueous solubility, poor bioavailability and pharmacokinetics, and rapid biotransformation under physiological conditions [28]. Several recent reviews have provided a comprehensive account of its pharmacological effects in cancer [18], diabetes mellitus, metabolic syndrome [29], and others [24, 30]. In this review, we will discuss recent progress of DG as a therapeutic agent against various neurological diseases along with its mechanisms of action in CNS. This review also critically evaluates existing limitations of DG solubility and bioavailability. It briefly recapitulates recent advances involving structural modification and formulations to increase its therapeutic efficacy.

## 2. Chemistry of Diosgenin

**Figure 1:**
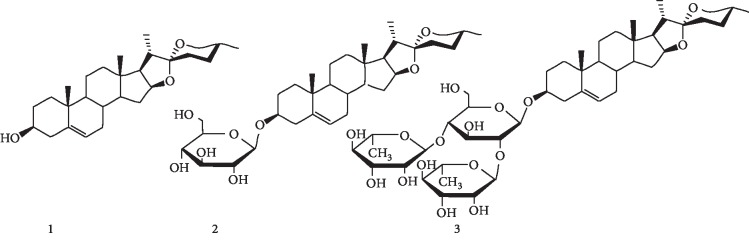
Structures of diosgenin (DG) and its glycosides.

Diosgenin (Figure 1, DG, 25*R*-spirost-en-3*β*-ol) is a C27 spiroketal steroid sapogenin belonging to a family of spirostanol steroidal compounds. Its molecular formula is C_27_H_42_O_3_ with a relative molecular mass of 414.62. DG is a white needle crystal or light amorphous powder with a proven thermal and chemical stability under various physical conditions [31]. DG is relatively stable against temperature and light exposure. However, DG is destabilized when it is exposed to hydrochloric acid [31]. DG is strongly hydrophobic (with Log *P* = 5.7), and it is insoluble in water [32, 33]. The solubility of DG is around 0.7 ng/mL in aqueous medium [34]. However, it is highly soluble in most nonpolar organic solvents (such as chloroform, dichloroethane, propanol, ethyl acetate, and propylacetate) and in partially polar solvents (such as acetone, methanol, and anhydrous ethanol).

## 3. Sources of Diosgenin

Primary sources of diosgenin (DG) include the *Dioscorea* species, *Heterosmilax* species, and *Trigonella foenum-graecum*, although DG and related steroidal sapogenins can be commercially obtained from tubers of various *Dioscorea* species [17]. DG is present in high levels in tubers of various wild yams (*D. villosa* Linn). A total of 137 types of *Dioscorea species* contain DG. Of them, 41 contain DG at more than 1%. The seeds of fenugreek (*T. foenum graecum* Linn) [35] and the rhizomes of *D. zingiberensis* are also important sources of DG. In addition, *Trillium govanianum* and *Costus speciosus* contain around 2.5% and more than 2.12% of DG, respectively [36–38]. DG is mainly generated by the hydrolysis of steroidal saponins in the presence of a strong acid, base, or enzyme catalyst [39]. Currently, microbial transformation is a promising method for the production of DG because of its environmentally friendly, highly specific, and mild reaction conditions at a low cost [36, 40].

## 4. Biosynthesis of Diosgenin

DG is biosynthesized from cholesterol via the isoprenoid pathway in several plant species [41, 42]. The biosynthesis of DG starts with acetyl CoA. It involves several steps to generate squalene that cyclizes to yield lanosterol. Lanosterol is further catalyzed to cholesterol by various enzymes. Cholesterol is sequentially converted to glucoside furostanols and spirostanols. These glycosides are eventually converted to spirostanols after the elimination of the glucose molecules at C26, resulting in ring closure during the catalysis of glucosidases. DG aglycone may convert to glycoside forms with mono-, di-, or trisaccharides known as saponins (Figure 1, Compounds **2** and **3**; Compound **3** is also called dioscin). The attachment of a carbohydrate moiety improves both the solubility and potency of DG.

## 5. Toxicity and Safety of Diosgenin

Diosgenin (DG) shows high biocompatibility and low toxicity. For instance, in an acute study, the oral administration of a single dose of 112.5–9000 mg/kg ethanol extracts of *Dioscorea* sp. containing 28.34% DG (31.7-2550.6 mg/kg) did not result in any signs of acute toxicity in rats. In a subchronic toxicity study, Sprague-Dawley rats orally administrated with DG at doses of 127.5, 255, and 510 mg/kg/day for 30 days did not show any significant changes in biochemical or hematological parameters. DG was the main metabolite in the serum [43, 44]. Additionally, a toxicological assay using *D. villosa* (DV) root extract showed that both acute (5 g/kg, single dose) and subchronic (1 g/kg/day, 30 days) treatments of rats resulted in only unremarkable changes in hematological, biochemical, and histopathological parameters [45]. Wojcikowski et al. reported that no acute renal or hepatic toxicity was observed with a crude extract of DG obtained from *D. villosa* at a dose of 0.79 g/kg/day administered orally. However, an increase in kidney fibrosis and liver inflammation was found when mice received a continuous treatment for 28 days in the same study. In a 90-day subchronic study, no toxic sign was found in mice fed fenugreek seeds at doses of DG ranging from 1% to 10% [46]. However, an *in vitro* study showed deleterious effects of DG mediated via genetic instability. At concentrations greater than 30 *μ*M, DG reduced cell viability and increased micronucleus frequency. It also has a significant cytostatic effect with DNA damage in HepG2 cells [47].

## 6. Bioavailability and Pharmacokinetic Studies of Diosgenin

Using a rat model, Okawara et al. have elucidated the pharmacological effect of cyclodextrin-bound diosgenin (DG). The peak level of DG in the skin was observed at 6 h after oral administration. The plasma concentration of an orally administered DG reached *C*_max_ (132.5 ± 48.2 ng/mL) at 5.01 ± 0.55 h with an AUC of 4121.9 ± 1354.7 ng · h/mL and an absolute oral bioavailability of 4.45 ± 1.46% [32, 33]. Similarly, Liu et al. have demonstrated that treatment with DG resulted in a *C*_max_ of 42.1 ± 30.4 ng/mL at 11.3 ± 3.9 h along with an AUC_0–60 h_ of 1309.3 ± 849.8 ng h/mL and *t*_1/2_ of 10.4 ± 4.2 h [48]. It was suggested that a single dosage of DG administered to rats, monkeys, and dogs was mostly excreted into feces, while the amount absorbed was rapidly eliminated via bile. Tissue distribution of DG in rats most notably occurred in the liver, adrenals, and gastrointestinal walls. Unchanged DG at concentrations up to 15 *μ*g/mL was found when multiple doses (100 mg/kg/day for 4 weeks) were administered to dogs. Several metabolites of DG were found in the bile of rats and dogs with a pattern of metabolites different in the two tested species. One of its major biliary metabolites was monohydroxylated diosgenin in the F ring. In humans, oral administration of DG at 3 g/day for 4 weeks did not alter the levels of DG in human serum (less than 1 mg/mL) [43].

## 7. Semisynthetic Derivatives of Diosgenin against Neurological Diseases

Although DG possesses numerous pharmacological activities against various diseases, it has weak biological activity, low aqueous solubility, poor pharmacokinetic profile, and instability under physiological conditions which greatly hinder its clinical application. Covalent modification of therapeutic agents is a clinically proven strategy that can enhance treatment efficacies. Semisynthetic modification of DG at C3 can address these issues by altering its physicochemical characteristics, thus improving its metabolic profile in terms of adsorption, distribution, metabolism, elimination, and biological activities.

**Figure 2:**
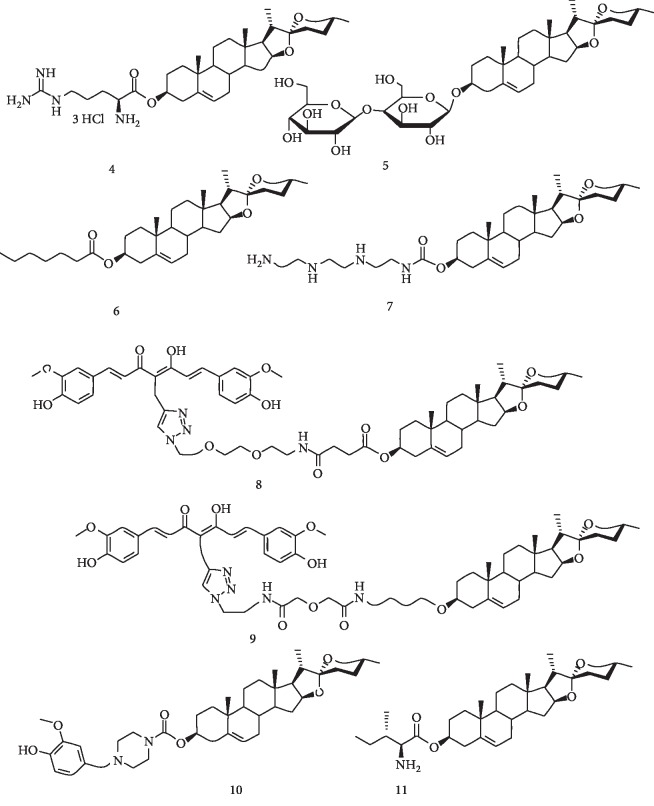
Chemical structures of semisynthetic diosgenin analogs.

To date, a variety of DG derivatives have been designed and synthesized, and most of them have shown improved physicochemical properties and enhanced pharmacological activities compared to parent drug DG. Semisynthetic DG derivatives can be roughly divided into four major categories on the basis of covalent linkage and attached functional entities. First, an amino acid prodrug strategy has been successfully used in the oral delivery of drugs that have low solubility and permeability [49, 50]. The introduction of an amino acid, either natural or its analog, to a parent drug generally can increase the aqueous solubility by orders of magnitude through an ionized carboxylate anion or the formation of amine salts. Moreover, various amino acid transporters are expressed in brush-border membranes of intestinal epithelial cells known to play a significant role in the absorption of several amino acid prodrugs [51]. In the past decade, a series of DG amino acid derivatives have been synthesized for the treatment of cancer, inflammation, diabetes, thrombosis, and neurodegenerative disorders [52]. Representative structures of diosgenin-arginine derivatives (Compound 4, Arg-DG) are presented in Figure 2 [50]. Second, the carbohydrate moiety plays a critical role in biological functions of steroidal saponins [53, 54]. An increasing number of synthetic steroidal saponins (DG-carbohydrates) have been created and tested for their biological activities mainly against inflammation and cancer (Compound 5) [55]. Third, DG-fatty acid derivatives with a hydrophobic moiety have been designed and prepared based on DG 3-caproate, also known as caprospinol, a naturally occurring compound in *Gynura japonica* that can protect neuronal cells from A*β*1–42 neurotoxicity [56]. Additionally, a series of DG derivatives with hydrophilic moieties such as PEG oligomers [28] and polyamines [57] have been synthesized to improve its solubility and biological activity (Compound 7). Fourth, DG-drug conjugates including DG moieties have been covalently linked to other therapeutic agents directly or via a linkage, forming codrugs with enhanced physicochemical, biopharmaceutical, and drug delivery properties (Compounds 8 and 9) [58, 59]. Several representatives of DG-drug conjugates reported in previous studies are presented in Figure 2.

## 8. Novel Formulations and Increased Bioavailability of Diosgenin

In addition to structural modification, drug delivery system, particularly nanotechnology, can be used to develop novel formulations with improved solubility, enhanced pharmacokinetics, and/or target delivery. Okawara et al. have prepared DG-cyclodextrin (CD) complexes to improve the skin concentration of DG and its pharmacokinetic profile, resulting in about 4- to 11-fold higher oral bioavailability of DG in the inclusion complex of DG/*β*-CD compared to a DG suspension [32, 33]. In addition, the bioavailability of DG has been further improved by combining *β*-CD and liquid crystal DG to enhance the bioavailability of poorly water-soluble drugs [34]. Recently, DG nanocrystals have been prepared via a media-milling method using a combination of pluronic F127 and sodium dodecyl sulfate as surface stabilizers, resulting in a significant improvement in the dissolution rate and pharmacokinetic profile compared to DG alone as well as increased bioavailability of DG [60]. An eight-arm-PEG-DG conjugate has been prepared for hydrophobic drug delivery via self-assembly to nanoparticles [61]. It has been reported that hyaluronate-DG conjugation via esterification can promote self-assembly into stable, negatively charged nanoparticles measuring 159-441 nm in water, which significantly enhances its solubility [62]. The DG-PEG conjugate can self-assemble into micelles in water, thus significantly enhancing the therapeutic efficacy for the prevention of arterial thrombus and venous thrombus [63].

## 9. Pharmacological Activity and Mechanism of Diosgenin and Its Derivatives in Central Nervous System (CNS) Diseases

The experimental design, pharmacological evidence, and underlying mechanism for diosgenin, dioscin, and diosgenin derivatives against various diseases in the central nervous system are summarized in Table 1.

### 9.1. Alzheimer's Disease and Parkinson's Diseases

Alzheimer's disease (AD) is one of the most common neurodegenerative disorders characterized by learning disabilities and declining cognitive function. It is a multifactorial disease caused by multiple etiological and pathogenic mechanisms. However, the exact mechanism underlying AD remains unclear. Several hypotheses such as amyloid-*β* (A*β*) accumulation, hyperphosphorylation of Tau, altered energy metabolism, oxidative stress, and neuroinflammation have been proposed [64].

Extracellular aggregation of A*β* leading to the formation of plaques via stepwise formation of oligomers and fibrils is a neuropathological hallmark of AD brains. Reduction in A*β* has been considered as a major therapeutic strategy against AD [65]. Tohda et al. have reported that DG can significantly improve memory loss and spike firing in the medial prefrontal cortex and hippocampal CA1 in 5XFAD mice. The accumulation of A*β* plaques and neurofibrillary tangles in the cerebral cortex and hippocampus was significantly decreased after DG treatment. Additionally, DG treatment decreased the number of degenerated axons and presynaptic terminals in regions surrounding amyloid plaques. These events were mediated by 1,25*D*_3_-membrane-associated, rapid response steroid-binding protein (1,25*D*_3_-MARRS) [66, 67], or heat shock cognate 70 by normalization of *α*-tubulin expression, which is a potentially critical event in axonal formation [25]. Koh et al. have reported that DG can ameliorate multiple types of brain injury in transgenic 2576 (TG) mouse models, in which the accumulation of A*β* plaques is induced by A*β*-42 peptides and neurotoxicant trimethyltin (TMT). Their results demonstrated that the numbers of A*β* plaques and dead cells in the granule cell layer of the dentate gyrus were significantly decreased by pretreatment with DG for 21 days. Additionally, a significant increase in the expression of nerve growth factor (NGF) and variation in corresponding components of NGF signaling pathways were found, suggesting that DG could stimulate NGF biosynthesis in multiple types of brain damage [68].

Although extracellular accumulation of neurotoxic A*β* species in the brain has been proposed as one of the main events in early stages of AD, continued failure of clinical trials involving A*β*-targeting drugs have prompted scientists to explore alternative mechanisms and therapeutic strategies [69]. Neurofibrillary tangles (NFTs) mainly composed of hyperphosphorylated Tau are another histopathological hallmark of AD and associated tauopathies. Hyperphosphorylation of Tau protein can lead to neurodegenerative disorders. Tohda et al. found that DG treatment can also decrease the hyperphosphorylation of Tau protein in the cortex and hippocampus in an AD mouse model [66, 67].

Teper et al. have identified the diosgenin analog (22*R*,25*R*)-20*α*-spirost-5-en-3*β*-yl hexanoate (Figure 2, diosgenin 3-caproate; caprospinol, Compound 6) using the 22*R*-hydroxycholesterol chemical structure as a probe. Compound 6 is a naturally occurring compound in *Gynura japonica*, a plant belonging to the Asteraceae family. It can protect neuronal PC12 cells against A*β*1-42-induced neurotoxicity [70]. In later studies, Lecanu et al. and Tillement et al. have screened potential candidates among the prepared DG derivatives in human NT2N neuronal cells and PC12 cells against *β*-amyloid (1-42)- (A*β*-) induced neurotoxicity. Their results showed that DG and its derivatives exert their neuroprotective effect via inhibiting the formation of neurotoxic amyloid-derived diffusible ligands and preferentially binding to two binding sites of A*β* identified by computational docking simulations in contrast to 22*R*-hydroxycholesterol that could only bind a single site. A subsequent study has revealed that Compound 6 has a direct effect on mitochondrial function. It blocked A*β* uptake by mitochondria in neuronal cells and protected SK-N-AS cells from A*β*-induced mitochondrial impairment by targeting Complexes 4 and 5 of the respiratory chain, indicating that DG derivatives might have potential in AD therapy [71, 72]. Compound 6 can prevent the formation of amyloid-derived diffusible ligands (ADDLs) by binding to A*β*42, decreasing amyloid accumulation in mitochondria, and directly targeting the mitochondrial respiratory chain [73]. Lecanu et al. have found that the neuroprotective effect of Compound 6 against AD is mediated by decreasing the level of tau phosphorylation [74]. Compound 6 can significantly reduce neurodegeneration and attenuate memory loss and cognitive disorders in a rat model of AD. The recovery from cognitive impairment is accompanied by a reduction in amyloid accumulation in the hippocampus [74]. A series of DG derivatives with different lengths of lateral carbon chains at C3 have been investigated. The structure-activity relationship has revealed that a six-carbon Compound 6 is the most effective one among all tested derivatives at different concentrations. The closest analogs with chain lengths of 4 to 5 carbons failed to exhibit any neuroprotective activity at the lowest concentration of 10 *μ*M, while the analogs with chains longer than C6 were less effective than Compound 6 [73]. Papadopoulos and Lecanu have reviewed the pharmacological activity of Compound 6 [56, 73].

Apart from the naturally occurring diosgenin derivatives such as Compound 6, a series of multifunctional, bivalent diosgenin-curcumin conjugates have been developed as neuroprotectants to combat AD [27, 58]. The results showed a clear structural preference for the introduction of the methylene carbon between diosgenin and curcumin. The conjugate with a spacer length of 17 atoms showed the highest protective activity in MC65 neuroblastoma cells and a decrease in neuroprotective activity was observed when the spacer length was extended to 28 atoms. The most potent Compounds 8 and 9 are presented in Figure 2. Their mechanism of action involves antioxidant activity and inhibitory effects on amyloid-*β* oligomer formation by directly binding to A*β* [58]. Additionally, DG can act as a membrane-anchoring moiety to improve the access to the cell membrane for the conjugates, suggesting that DG is a novel anchor that can facilitate the multifunctional role of bivalent conjugates for further development as potential therapeutic agents for AD treatment [27]. Recently, Yang et al. synthesized a series of multifunctional DG derivatives and evaluated their effect from A*β*-induced damage in PC12 cells and improved learning and memory impairments in A*β*-injected mice. Among them, Compound 10 (AA36) significantly prevented A*β*-induced PC12 cell damage and restored the cognitive impairment in A*β*-injected mice, suggesting that DG is a promising skeleton structure for anti-AD drug development [59].

Parkinson's disease (PD) is another neurodegenerative disorder characterized by the loss of dopaminergic neurons in the substantia nigra. The protective effect of DG against LPS-induced PD has been evaluated using a rat model. DG can attenuate LPS-induced motor deficits in rats by suppressing the TLR4/NF-*κ*B signaling pathway [26]. Recently, Cai et al. has synthesized and evaluated the neuroprotective potential of the diosgenin-amino acid derivative and the diosgenin derivative conjugated with L-isoleucine (Compound 11) displayed a neuroprotective role on damaged SH-SY5Ycells by reducing apoptosis as well as promoting angiogenesis at 4 mg/mL on the chorioallantoic membrane model [75].

### 9.2. Cognitive Effects

Oxidative stress is intimately associated with cognitive function in neurodegenerative disorders such as Alzheimer's disease. Chiu et al. have acknowledged the neuroprotective effects of DG-rich yam in senescent mice induced by D-galactose (D-gal). Compared with D-gal treatment alone, DG treatment for 4 weeks significantly restored learning and memory impairment in mice starting from week six. The mechanism is partly mediated through an increase in endogenous antioxidant enzyme activities [76]. They found similar results after treatment with DG at the concentration range of 5 to 125 mg/kg [77]. Turchan-Cholewo et al. have reported that DG may improve the cognitive impairment associated with human immunodeficiency virus (HIV) infection. Increased levels of oxidative stress and the E4 allele of apolipoprotein E (ApoE) have been found in the CNS of an HIV-infected population or in individuals with a history of intravenous drug abuse, and were considered as the risk factors contributing to the development of dementia. The results revealed that HIV proteins such as gp120, Tat, and Tat+morphine treatment increased the neurotoxicity in cultured human neuronal cells with ApoE4. DG can protect against neurotoxicity induced by Tat+morphine treatment, and the Tat-induced oxidative stress-impaired morphine metabolism can be prevented by DG treatment [78]. Moreover, the learning and memory capacity of Compound 2 derived from *Trillium tschonoskii* Maxim has been investigated in aging rats induced by D-gal with impaired cognitive function using the Morris water maze test. Treatment with Compound 2 improved the learning and memory capacity in aging rats induced by D-gal and the mechanism might be associated with rescuing dysfunctional autophagy via the upregulation of Rheb and the downregulation of mTOR signaling, suggesting that Compound 2 has potential in health promotion and aging-related disease therapy [79].

### 9.3. Neuroinflammation

Overactivated microglia are present in large numbers in several neurodegenerative disorders. Overproduction of various proinflammatory cytokines may result in neurotoxicity in neurodegenerative disorders. Cumulative evidence has suggested that microglial activation is an early and ongoing stage in neurodegenerative disorders. Anti-inflammatory drugs might be beneficial during the early stages of diseases in several animal models. They can inhibit microglial activation and result in the suppression of proinflammatory cytokines in the hippocampus, finally reversing the decline in memory and learning. It is a practical strategy to develop therapies by preventing the progression of neurodegenerative disorders via the modulation of the neuroinflammation markers in the hippocampus [80].

Binesh et al. have investigated the efficacy of DG in the amelioration of atherosclerotic progression in the heart and inhibition of inflammatory mediators in the liver and brain of Wistar rats treated with an atherogenic diet. DG can inhibit the inflammatory mediators triggered by atherogenic diet in the heart, liver, and brain of rats via the downregulation of COX-2, TNF-*α*, and NF-*κ*Bp65, thereby preventing atherosclerotic disease progression [81]. Yang et al. have reported that Compound 3 can rescue endotoxemia-induced neuroinflammation and has a neuroprotective effect on hippocampal neurogenesis impaired by neuroinflammation, which is consistent with the results obtained in the behavior test showing that Compound 3 reversed cognitive impairment. The endotoxemia-triggered neuroinflammation cascade involves the neurotransmitter 5-HT and the HMGB-1/TLR4 signaling pathway [82]. Wang et al. has reported that Compound 2 extracted from *Tritulus terrestris* L. can selectively inhibit inflammatory M1 markers (NO, IL-6, and TNF-*α*) in activated rat microglia and BV2 cells induced by LPS, without affecting the production of inflammatory M2 markers (IL-10, IL-1R*α*, and CD206) in LPS- and IL-4-treated microglia. Its mechanism involves the inactivation of NF-*κ*B, ERK1/2/MAPK, and p38/MAPK signaling pathways indicating that DG glycoside might be a potential candidate for the treatment of various neurodegenerative disorders mediated by neuroinflammation [83].

Recently, we reported that DG derivatives carrying primary amine (Compound 7, DGP) or the amino acid L-arginine (Compound 4, Arg-DG) at the C3 show a significant increase in aqueous solubility and anti-inflammatory activity in LPS-induced BV2 cells compared to DG. The possible mechanisms of both derivatives involve the inhibition of NF-*κ*B activation and JNK/MAPK signaling [57, 84]. Arg-DG can also rescue hippocampal neurogenesis and cognitive function impaired by LPS via the inhibition of microglial overactivation, the expression of the TLR4 receptor and downstream signaling of NF-*κ*B and JNK/MAPK, and the ultimate suppression of proinflammatory cytokines. Our results suggested that the chemical modification of DG might be an effective approach to improve its physicochemical properties and pharmacological activities in neurodegenerative disorders resulting from microglia-mediated neuroinflammation [84]. Interestingly, although the DG glycoside and DG derivatives including DGP and Arg-DG exhibited strong anti-inflammatory activity in LPS-induced microglial BV2 cells, they differed in inhibitory activity against MAPK signaling. Structural differences involving the substituted groups of DG at C3 might have led to the selective inhibition of MAPK subfamily members (ERK1/2, JNK, and p38).

### 9.4. Multiple Sclerosis

Multiple sclerosis (MS) is a chronic inflammatory demyelinating disease involving the central nervous system. Currently, curative drugs are unavailable for MS in clinics. Phagocytosis by microglia or macrophages is considered a hallmark of MS lesions. Activated microglia with different phenotypes exhibit either neuroprotective or neurotoxic effects in MS depending on the disease stage and severity of disease. They might lead to a relapsing-remitting MS [80, 85].

Recently, Liu et al. have reported the therapeutic potential of DG in an experimental autoimmune encephalomyelitis (EAE) model of mice using myelin oligodendrocyte glycoprotein. Their results showed that DG significantly alleviated the progression of EAE in mice and obviously reduced the inflammation and demyelination in the CNS. A mechanistic study has shown that DG can inhibit the microglial/macrophage activation, reduce CD4^+^ T cell proliferation, and suppress Th1/Th17 cell differentiation [86]. Additionally, in their earlier study involving a purified rat OPC culture model, DG significantly and specifically promoted the differentiation of oligodendrocyte progenitor cells (OPCs) into mature oligodendrocytes, which is considered as a prerequisite for remyelination after demyelination. Moreover, DG administration can enhance remyelination in a demyelination model induced by cuprizone. DG can also significantly increase the number of mature oligodendrocytes in the corpus callosum without affecting the number of OPCs. The underlying mechanism for the accelerated remyelination is attributed to the ER-mediated ERK1/2 activation. DG not only attenuates the progression of EAE, and reduces demyelination and inflammation of CNS as an immunomodulator, but also promotes the differentiation of OPCs, and enhance remyelination and the number of OPCs in demyelinating lesions of the CNS, suggesting that DG is a promising therapeutic candidate in the treatment of MS [87].

### 9.5. Spinal Cord Injury

Spinal cord injury (SCI) is a severe neurological disorder of CNS that usually causes permanent disability or motor deficit and sensory loss in patients, and it also leads to numerous complications associated with complex pathological mechanisms. Very few restorative therapeutic options are available clinically to improve the neurologic deficits in the SCI. Chen et al. has reported that the DG-rich extract of *Trillium tschonoskii* Max has a neuroprotective role against the spinal cord in rats by upregulating the expression of ciliary neurotrophic factor (CNTF) and its receptor (CNTFR*α*) at mRNA and protein levels [88]. Chen et al. has further evaluated the neuroprotective effect of the bioactive component of Compound 2 in *T. tschonoskii* Max on motor function recovery and the underlying mechanism after SCI in rats. Compound 2 could significantly reduce tissue injury and edema. The underlying mechanism might be associated with autophagy via the suppression of p62 expression and upregulation of Rheb/mTOR signaling due to the downregulation of miR-155-3p, leading to the prevention of neuronal cell damage and apoptosis [89].

### 9.6. Stroke and Thrombosis

Stroke is a major public health concern with high morbidity and mortality. The main stroke pathological types include ischemic stroke, primary intracerebral hemorrhage, and subarachnoid hemorrhage. Stroke is the third leading cause of death worldwide with a progressively increasing incidence and involving younger individuals. Thus, developing therapies to treat stroke is highly desirable [90]. Zhu et al. have reported that Compound 3 has therapeutic potential against ischemic stroke in rats. Compound 3 can significantly reduce the infarct volume and neurological scores in rat models of ischemic stroke. Compound 3 can inhibit the expression levels of TLR4, myeloid differentiation factor 88 (MyD88), and activation of NF-*κ*B, leading to the inhibition of inflammatory responses in a rat model of ischemic stroke, suggesting that Compound 3 acts as an anti-inflammatory agent against ischemic stroke via the inhibition of the TLR4/MyD88/NF-*κ*B signaling pathway [91].

Thrombosis of cerebral arteries is the major cause of morbidity and mortality worldwide. Zhang et al. have designed several monomers of diosgenyl saponin using a simple and convenient method. The antithrombotic effects of a synthetic disaccharide saponin of DG attached to two glucose units (Compound 5) along with the naturally occurring DG saponin have been examined. Their results showed that Compound 5 exhibited a strong efficiency in prolonging bleeding time and altering platelet aggregation both *in vitro* and *in vivo*. Moreover, their results demonstrated that Compound 5 could inhibit platelet aggregation, prolong the activated partial thromboplastin time, reduce factor VIII activities in rats, and significantly enhance the protection in mice. However, its mechanism of action was not determined in their study [55]. It needs to be examined in further studies.

### 9.7. Cerebral Brain Ischemia-Reperfusion Injury

Cerebral I/R injury refers to cerebral ischemia-induced brain damage that occurs if blood supply is restored [92]. It often causes a series of consequences including neurotoxicity induced by excitatory amino acids (EAA), mitochondrial dysfunction, overproduction of reactive oxygen species (ROS), inflammatory reaction, and neuronal cell death, which ultimately leads to irreversible brain injury. Great strides have been made in treatment modalities for ischemic stroke. However, an effective therapeutic strategy is currently unavailable for ischemic stroke clinically. Ischemic stroke is still a major cause of deaths in developed countries [93]. A recent study has uncovered that DG is effective in treating transient cerebral I/R injury via different mechanisms. Intragastric administration of DG once daily for 7 days prior to surgery can significantly inhibit the death rate in rats, restore motor impairment, and reduce neurological deficit scores along with cerebral infarct size. DG decreased the cellular apoptosis in the hippocampus CA1 and cortex via suppression of caspase-3 activity and Bax/Bcl-2 ratio. Moreover, DG can suppress the overproduction of proinflammatory cytokines including TNF-*α*, IL-1*β*, and IL-6 in the blood serum of I/R insulted rats. Its action is mediated by blocking the NF-*κ*B signaling pathway via the downregulation of I*κ*B*α*, suggesting that DG has a great potential to combat similar diseases in the clinic [94].

Compound 3 (Figure 1), a saponin of diosgenin, also possesses neuroprotective activities against ischemia-reperfusion injury. It can protect PC12 cells and primary cortical neurons in oxygen-glucose deprivation and reoxygenation (OGD/R) insults *in vitro*. It can also significantly attenuate cerebral I/R injury in the middle cerebral artery occlusion (MCAO) model. Further studies have indicated that the neuroprotective mechanism of Compound 3 is related to a blockade of the HMGB-1/TLR4/MyD88/TRAF6 signaling pathway via the inhibition of transcriptional activities of NF-*κ*B and AP-1, the phosphorylation of MAPK and STAT3, and the proinflammatory cytokine responses and augmentation of anti-inflammatory mediator levels. These findings indicate that Compound 3 is a potential therapeutic agent for the prevention of cerebral I/R injury [95]. Furthermore, a combination treatment of Compound 3 and baicalein has resulted in a significant improvement in spatial memory and reduction in the infarct volume in a mice model of cerebral I/R injury. Hippocampal gene expression profiles of MCAO ischemic mice using cDNA microarray analysis of 1176 known genes have shown that numerous genes including those involved in cell cycle regulation, DNA binding, signal transduction pathways, and neuronal transcription factors are associated with neuroprotective effects [96].

### 9.8. Antidepressant Effects

Depression is becoming a common neuropsychiatric disorder. However, there is no effective antidepression therapy available clinically. Ho et al. reported that chronic diosgenin administration at a dosage of 10 mg/kg/day can improve avoidance behavior in a learned helplessness test involving ovariectomized rats with high anxiety levels but not in low-anxiety rats. Chronic administration of DG can reduce the expression of IL-2, an indicator of neuroimmune function, in the brains of ovariectomized rats, suggesting that DG might relieve depressive behavior via the modulation of the neuroimmune system [97]. Moreover, Yang et al. have reported that Compound 3 exhibits the antidepressant effect by enhancing 5-HT levels in endotoxemia-induced acute neuroinflammation in mice [82].

### 9.9. Neuropathic Pain

Neuropathic pain is a prevalent and complicated condition arising from diseases such as diabetes mellitus (DM), postherpetic neuralgia, and brain injury affecting the peripheral or central nervous system. It is often resistant to treatment. It is associated with poor treatment satisfaction in patients [98]. Zhao et al. has shown the effect of DG on allodynia and the underlying mechanism in a neuropathic pain model of rats induced by chronic constriction injury (CCI). Their results showed that DG could significantly reverse mechanical allodynia and thermal hyperalgesia induced by CCI. DG can alleviate CCI-induced neuropathic pain in rats by inhibiting the activation of the p38MAPK and NF-*κ*B pathways, ultimately leading to the suppression of CCI-induced overexpression of proinflammatory cytokines including tumor necrosis factor-*α* (TNF-*α*), interleukin- (IL-) 1*β*, and IL-2, and reduction of oxidative stress induced by CCI [99]. In addition, Zhao et al. has shown the efficacy of DG in the alleviation of neuropathic pain in streptozotocin- (STZ-) induced diabetic rats. Their study revealed that daily administration of DG at a dose of 40 mg/kg over 5 weeks obviously increased the mechanical and thermal nociceptive thresholds and lowered the pain score at delayed stages of the formalin test, but not in the early stage. An antinociceptive mechanism of DG is that it can lower oxidative stress and inflammation in diabetic rats via the restoration of the malondialdehyde (MDA) level, the activity of superoxide dismutase (SOD) and catalase, and the expression of TNF-*α* and IL-1*β* via NF-*κ*B signaling [100]. Moreover, Kang et al. has reported that DG from *Dioscorea nipponica* can ameliorate diabetic neuropathy in diabetic rats. DG can increase levels of the nerve growth factor (NGF) that are reduced in diabetic rats and enhanced nerve conduction velocities in a mouse model of diabetic neuropathy. Additionally, DG can increase neurite outgrowth in PC12 cells, improve damaged axons, reduce disorganization of the myelin sheath and increase the area of myelinated axons. Beneficial effects of DG in a diabetic neuropathy model in restoring ultrastructural changes and neural regeneration might be associated with increased expression of NGF [101].

Lee et al. have examined the effect of DG on chronic pain and functional deficit resulting from sciatic crushed nerve injury in rats. DG treatment increased the sciatic function index and suppressed the nerve injury-induced overexpression of BDNF, TrkB, COX-2, iNOS, and c-Fos in the ventrolateral periaqueductal gray and paraventricular nucleus, suggesting that DG treatment could prolong pain control and extended functional recovery after peripheral nerve injury [102].

### 9.10. Glioblastoma

Glioblastoma is the most aggressive and malignant primary central nervous system cancer, characterized by rapid proliferation and high invasion. Surgical resection is still the mainstay of treatment for glioblastoma. Currently, no effective treatments are available to cure patients with glioblastoma due to its exceptionally heterogeneous nature and unique microenvironment. Temozolomide (TMZ) and bevacizumab are the only FDA-approved therapeutic agents for the treatment of primary and recurrent glioblastoma, respectively [105]. Acquired TMZ resistance seriously restricts the therapeutic index and fails to prolong the overall survival. DG can significantly reduce the dosage regimen of TMZ in the combinatorial therapy of DG and TMZ. It can also overcome TMZ resistance in glioblastoma cells as well. The underlying mechanism involves the downregulation of matrix metalloproteinase-2 (MMP-2) and the promotion of apoptosis [103]. The antitumor activity and the underlying mechanism of dioscin has been examined both *in vitro* and *in vivo*. Dioscin exhibited a growth inhibitory effect on C6 glioma cancer cells. It significantly inhibited tumor size and prolonged the life cycle of rats. The mechanism of action of dioscin involves the promotion of ROS accumulation, DNA damage, and mitochondrial-mediated apoptosis signaling [104].

### 9.11. Clinical Studies

Tohda et al. have investigated the effects of a DG-rich yam extract on cognitive enhancement in 28 healthy volunteers aged between 20 and 81 years recruited from the Toyama Prefecture, Japan. The administration of DG-rich yam extract for 12 weeks significantly improved the semantic fluency without any adverse effects, indicating that DG could enhance the cognitive function in healthy adults [106].

## 10. Conclusion and Future Perspectives

Diosgenin and its derivatives have attracted considerable attention from researchers worldwide. Several studies have described the pharmacological effects of DG and its derivatives against a variety of diseases such as cancer, diabetes, osteoporosis, AD, and stroke. Several reviews have emphasized the pharmacological advances of DG in the treatment of cancer and described the analytical methodology. In recent years, increasing experimental evidence has shown that DG and its derivatives exhibit promising therapeutic potential in several neurodegenerative and neurological disorders. Therefore, the present review mainly addressed recent progresses of DG and its potent derivatives against multiple diseases of CNS including AD, PD, stroke, neuroinflammation, multiple sclerosis, spinal cord injury, ischemia-induced brain damage, depressive disorders, neuropathic pain, glioblastoma, and cognitive impairment, along with their underlying mechanisms of action at the molecular and cellular levels. This review will facilitate the exploration of new horizons for further research of DG or its derivatives at the preclinical and clinical levels for potential treatment of neurodegenerative disorders.

Although DG is abundant in nature, with high biocompatibility and with thousands of reports elaborating the remarkable pharmacological properties of DG and its derivatives documented in the literature, most of the current results are derived from *in vitro* or animal studies which prevents definitive conclusions about its clinical efficacy. Clinical testing and validation of preclinical data are still insufficient, especially in the treatment of specific neurological disorders. Additional data from clinical trials are highly desired, including test period, dosage, formulation, ethical approvals, adverse effects, drug interactions, and food interactions. Additionally, most patients suffering from neurodegenerative disorders require lifelong medication due to the slow progression of such diseases. Therefore, a systematic experimental design to assess the long-term outcomes of DG and/or its derivatives for the treatment of neurodegenerative disorders and the management of related symptoms is highly recommended in future studies. Furthermore, the risk assessment and safety evaluation of the pharmacological use of DG or its derivatives in the treatment of neurodegenerative disorders need to be investigated in depth.

The successful development of a therapeutic candidate against neurological disorders is a challenge. First, DG suffers from several drawbacks including low solubility and poor pharmacokinetic profiles which severely restrict its clinical application. Structural modifications or drug delivery systems are reliable techniques to solve these limitations. For structural modification, a balanced analysis of biological activity, solubility, cytotoxicity, and the permeability of blood-brain barrier after modification should be conducted to screen for potential lead compounds for further study. Second, the pathogenesis of neurodegenerative diseases such as AD and PD is complex and multifactorial. The prevention and treatment of those diseases using DG or its derivatives alone might be unsatisfactory. Combination therapies of DG with compounds possessing multiple mechanisms of action are expected to be more effective than individual drugs to treat varied pathological aspects of these diseases. Further, a multitarget drug strategy against multiple risk factors in the development of therapies for neurodegenerative disorders is an essential paradigm and an innovative approach to treat neurological diseases with complex pathogenesis. Numerous studies have indicated that the multifunctional compounds can enhance therapeutic effectiveness and minimize side effects, subsequently leading to better patient compliance via simultaneous modulation of multiple targets in a selective manner.

## Figures and Tables

**Table 1:** Key pharmacological effects and mechanisms of action of DG and its major derivatives in neurological diseases.

Entry	Active ingredient	Experimental model	Pharmacological effect	Mechanisms of action	Ref
1	Diosgenin	5XFAD transgenic mouse model of AD; Rat cortical neurons and mouse cortical neuron primary culture	Increased memory and decreased axonal degeneration; Reduced amyloid plaques and neurofibrillary tangles in the cerebral cortex and hippocampus	1,25*D*₃-membrane-associated, rapid response steroid-binding protein (1,25*D*_3_-MARRS)	[65]
2	Diosgenin	Normal mouse	Improved memory and axonal density; Increased c-Fos expression in the medial prefrontal and perirhinal cortices	1,25*D*_3_-MARRS-triggered axonal growth	[66]
3	Diosgenin	Trimethyltin- (TMT-) injected transgenic 2576 (TG) mice	Decreased the number of A*β*-stained plaques and dead cells in the granule cell layer of the dentate gyrus; Reduced acetylcholinesterase (AChE) activity and Bax/Bcl-2 expression; increased expression of nerve growth factor (NGF) and superoxide dismutase (SOD) activity	Increased phosphorylation of downstream members in TrkA signaling; Evaluated p75(NTR) expression and JNK phosphorylation in the NGF signaling pathway	[67]
4	Compound 6	Memory-impaired Long-Evans rats induced by infusion of Fe^2+^, A*β*42, and buthionine-sulfoximine (FAB) into the left cerebral ventricle for 4 weeks	Enhanced cognitive function; Decreased amyloid deposits, astrogliosis, and Tau protein phosphorylation in hippocampus		[68]
5	Compound 6	A*β*-induced neurotoxicity in rat PC12 and human NT2N neuronal cells	Protected against 0.1 *μ*M A*β* in PC12 cells; Reversed 0.1-10 *μ*M A*β*-induced decrease in ATP levels	Physicochemical interaction with A*β* inhibited the formation of neurotoxic amyloid-derived diffusible ligands	[70, 71]
6	Compound 6	A*β*1-42-induced SK-N-AS cells	Protected MPT and inhibited accumulation of the A*β*1-42 in the mitochondrial matrix	Directly targeting complexes IV and the mitochondrial respiratory chain	[72]
7	Compound 8	A cellular AD model using MC65 neuroblastoma cells from TC withdrawal-induced cytotoxicity	Antioxidative ability and inhibitory effects on amyloid-*β* oligomer (A*β*O) formation	Bind directly to A*β*	[56]
8	Compound 9	A cellular AD model using MC65 neuroblastoma cells from TC withdrawal-induced cytotoxicity; Neuronal N2a cells and rat primary cortical neurons	Significant stimulating activity on neurotic outgrowth and the state 3 oxidative rate of glutamate while preserving the coupling capacity of the mitochondria	Interfere with glutamate uptake or its redox reaction	[27]
9	Diosgenin-rich yam extracts	Senescent mice induced by D-galactose	Improve their learning and memory abilities; Increase the activities of superoxide dismutase (SOD) and glutathione peroxidase (GPx) and decrease malondialdehyde (MDA) level	Enhancing endogenous antioxidant enzymatic activities	[56, 58]
10	Diosgenin	PD model using Sprague-Dawley rats using intrastriatal injection of lipopolysaccharide (LPS)	Attenuate the inflammatory and oxidative stress response; Restore LPS-induced motor deficits; Decrease the expression levels of TLR2, TLR4, and NF-*κ*B	Inhibiting the TLR/NF-*κ*B pathway	[26]
11	Diosgenin	*In vitro* model of HIV-induced dementia using human neuronal cultures with E4 allele of ApoE	Protected against the neurotoxicity of Tat+morphine; Tat-induced oxidative stress impaired morphine metabolism		[76]
12	Diosgenin	A rat model with brain aging through subcutaneous injection of D-galactose	Improve learning and memory; Upregulating Rheb and downregulating mTOR	Rescuing dysfunctional autophagy mediated by Rheb-mTOR signal pathway	
13	Compound 3	Neuroinflammation induced by intraperitoneal injection of LPS	Enhanced the serotonergic system and produced the antidepressant effect	Protects the hippocampus from LPS-induced neuroinflammation by the neurotransmitter 5-HT and the HMGB-1/TLR4 signaling pathway	[80]
14	Compound 2	Neuroinflammation model using rat microglia and BV2 cells induced by LPS	Suppressed the expression levels of proinflammatory M1 markers, such as NO, IL-6, and TNF-*α*; Repressed I*κ*B-*α*, ERK, MAPK, and p38 MAPK phosphorylation	Inhibiting NF-*κ*B, ERK/MAP, and p38/MAPK signaling	[81]
15	Compound 7	Neuroinflammation model using BV2 cells induced by LPS	Inhibition of the inflammatory mediators such as NO, iNOS, COX-2, IL-6/1b, and TNF-*α* in protein and mRNA levels; Suppressed the NF-*κ*B activity and phosphorylation level of JNK	Inactivation of NF-*κ*B and JNK MAPK signaling	[82]
16	Compound 4	Neuroinflammation model using BV2 cells or mice by I.C.V. injection of LPS	Improved the cognitive function impaired by LPS and attenuated LPS-impaired neurogenesis; Suppressed the production of proinflammatory cytokines in hippocampal DG	Blocking microglial activation; Underlying NF-*κ*B and JNK MAPK; Signaling in LPS-induced adult mice	[57]
17	Diosgenin	C57BL/6J mice model of experimental autoimmune encephalomyelitis	Inhibit the activation of microglia and macrophages, suppress CD4^+^ T cell proliferation, and hinder Th1/Th17 cell differentiation		[84]
18	Diosgenin	Rat primary oligodendrocyte progenitor cell (OPC) culture model, a cuprizone-induced demyelination C57BL/6J mice model	Significantly and specifically promotes OPC differentiation; Enhances remyelination; Increases the number of mature oligodendrocytes in the corpus callosum	Differentiation of OPC into mature oligodendrocytes through an ER-mediated ERK1/2 activation pathway to accelerate remyelination	[85]
19	Compound 2	Sprague-Dawley rats with traumatic spinal cord injury	Significantly less tissue injury and edema; Functional recovery	Significantly attenuated p62 expression and upregulated the Rheb/mTOR signaling pathway due to the downregulation of miR-155-3p	[87]
20	Compound 3	Ischemic stroke rat model	Improved infarct volume and neurological scores; Reduced inflammatory responses, and suppressed the expression of TLR4, MyD88, NF-*κ*B, TGF-*β*1, HMGB-1, IRAK1, and TRAF6	Inhibition of TLR4/MyD88/NF-*κ*B induced inflammation	[89]
21	Compound 5	Thrombosis model using male balb/C mice	Prolonging the bleeding time; Inhibited platelet aggregation, prolonged partial thromboplastin time (APTT), and inhibited factor VIII activities		[90]
22	Diosgenin	Transient focal cerebral ischemia-reperfusion (I/R) injury model by middle cerebral artery occlusion (MCAO) using the intraluminal thread for 90 min	Inhibited the death rate and improved the impaired neurological functions, neurological deficit scores, and cerebral infarct size; Reduced cell apoptosis in the hippocampus CA1 and cortex; Suppressed the production of proinflammatory cytokines TNF-*α*, IL-1*β*, and IL-6 in blood serum	Antiapoptosis, anti-inflammation, and intervening NF-*κ*B signaling pathway	[92]
23	Compound 3	*In vitro* oxygen-glucose deprivation and reoxygenation (OGD/R) model and an *in vivo* middle cerebral artery occlusion (MCAO) model	Prevented OGD/R insult and cerebral I/R injury; Inhibition in the expression and the nuclear-to-cytosolic translocation of HMGB-1; Blockade of the TLR4/MyD88/TRAF6 signaling pathway; Inhibited NF-*κ*B and AP-1 transcriptional activities, inhibited MAPK and STAT3 phosphorylation, inhibited proinflammatory cytokine responses, and upregulated the levels of anti-inflammatory factors	HMGB-1/TLR4 signaling	[93]
24	Compound 3	Cerebral ischemia-reperfusion model by middle cerebral artery occlusion (MCAO) ischemic mice	Enhanced spatial learning memory in ischemic mice; An improvement in deficient ability and reduction in infarct volume		[94]
25	Diosgenin	Ovariectomized (OVX) female Wistar rats	Dose-dependently influences IL-2 levels in the brain of OVX rats and affects depressive behavior in OVX with high-anxiety rats		[95]
26	Diosgenin	Neuropathic pain model induced by chronic constriction injury (CCI) in rats	Reversed the mechanical withdrawal threshold and thermal withdrawal latency; Inhibited the expression levels of proinflammatory cytokines TNF-*α*, IL-1*β*, and IL-2; Suppressed oxidative stress	Inhibiting activation of p38 MAPK and NF-*κ*B signaling pathways	[96]
27	Diosgenin	Diabetic neuropathy mice model	Increased NGF levels in the sciatic nerve, enhanced neurite outgrowth in PC12 cells, and improved nerve conduction velocities; Reduced disarrangement of the myelin sheath, increased area of myelinated axons, and an improvement in the damaged axons	Increased the nerve conduction velocity by induction of NGF	[97]
28	Diosgenin	Peripheral nerve injury model using male Sprague-Dawley rat to crush the right sciatic nerve for 30 sec	Increased sciatic function index (SFI) value; Suppressed nerve injury-induced c-Fos expression in the ventrolateral periaqueductal gray (vlPAG) and paraventricular nucleus (PVN); Increased expression levels of BDNF, TrkB, COX-2, and iNOS		[99]
29	Diosgenin	C6 rat glioma cells	Reduced the dosage regimen of TMZ and overcome temozolomide resistance in TMZ-resistant GBM cells; Underwent apoptosis and early cell cycle arrest with significant reduction in MMP-2 levels	Upregulation of MMP-2 level and apoptosis signaling pathway	[103]
30	Compound 3	*In vitro* study using GBM, U87MG, A172, LN18, NBRC, T98G, and LN229 cell lines	Inhibited proliferation of C6 glioma cells, ROS generation caused mitochondrial damage and cell apoptosis; Inhibited tumor size and extended the life cycle of rats	Increase in ROS accumulation, DNA damage, and mitochondrial-mediated apoptosis signaling	[104]
